# Supplementary figures and images for: Forecasting national and regional influenza-like illness for the USA
Source: PLoS Comput Biol. 2019 May 23;15(5):e1007013. doi: 10.1371/journal.pcbi.1007013 (PMC6557527; doi:10.1371/journal.pcbi.1007013)

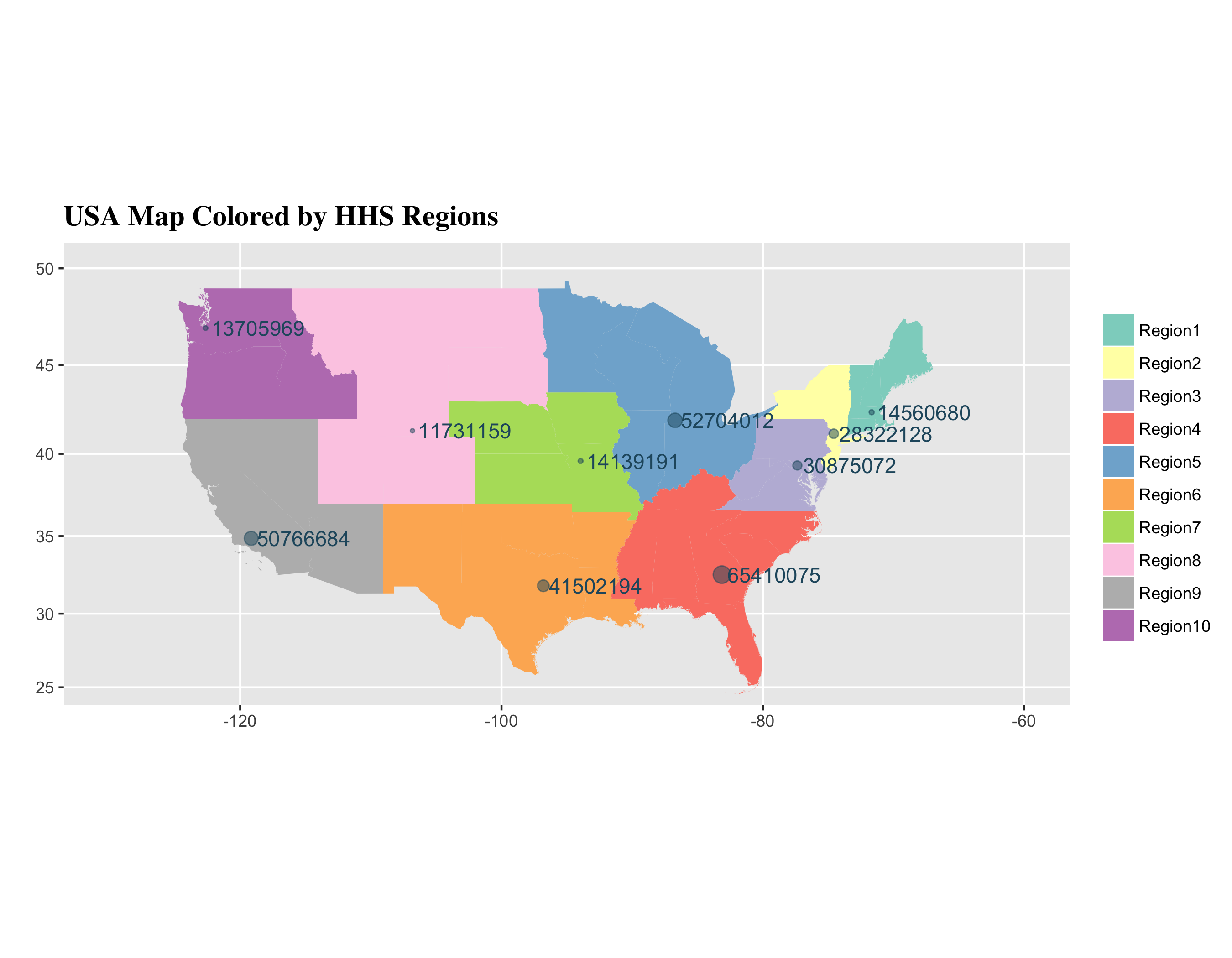

Supplement: S1 Fig — The green circle in each HHS region denotes the population density weighted location of the centroid of the region, and the radius of each circle is proportional to the weight of the region which is determined by its relative population. The population of each region is denoted on the map next to the centroid. (PNG) [file pcbi.1007013.s001.png]

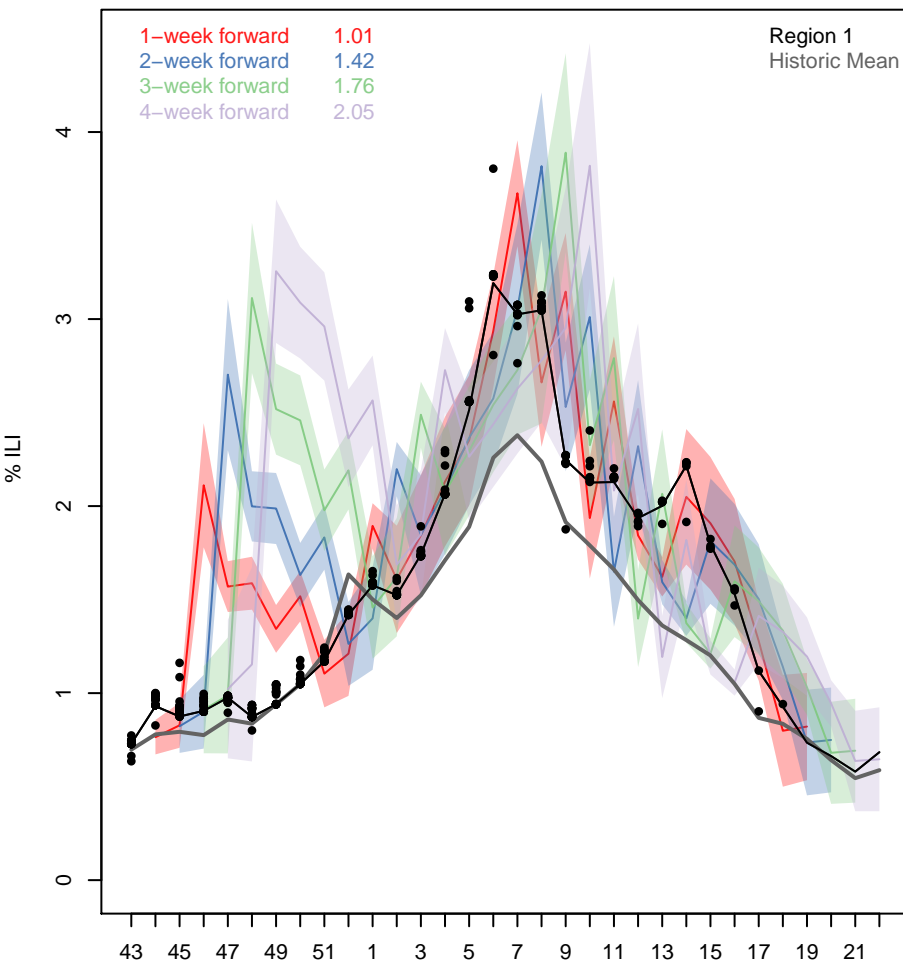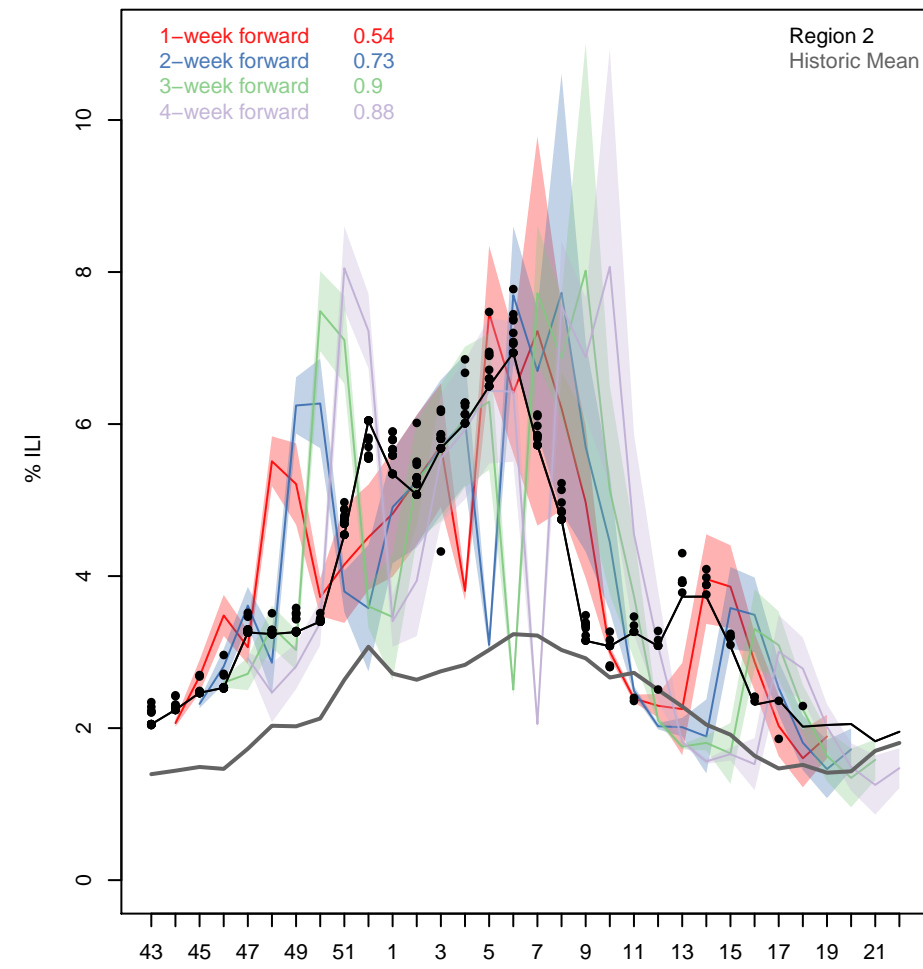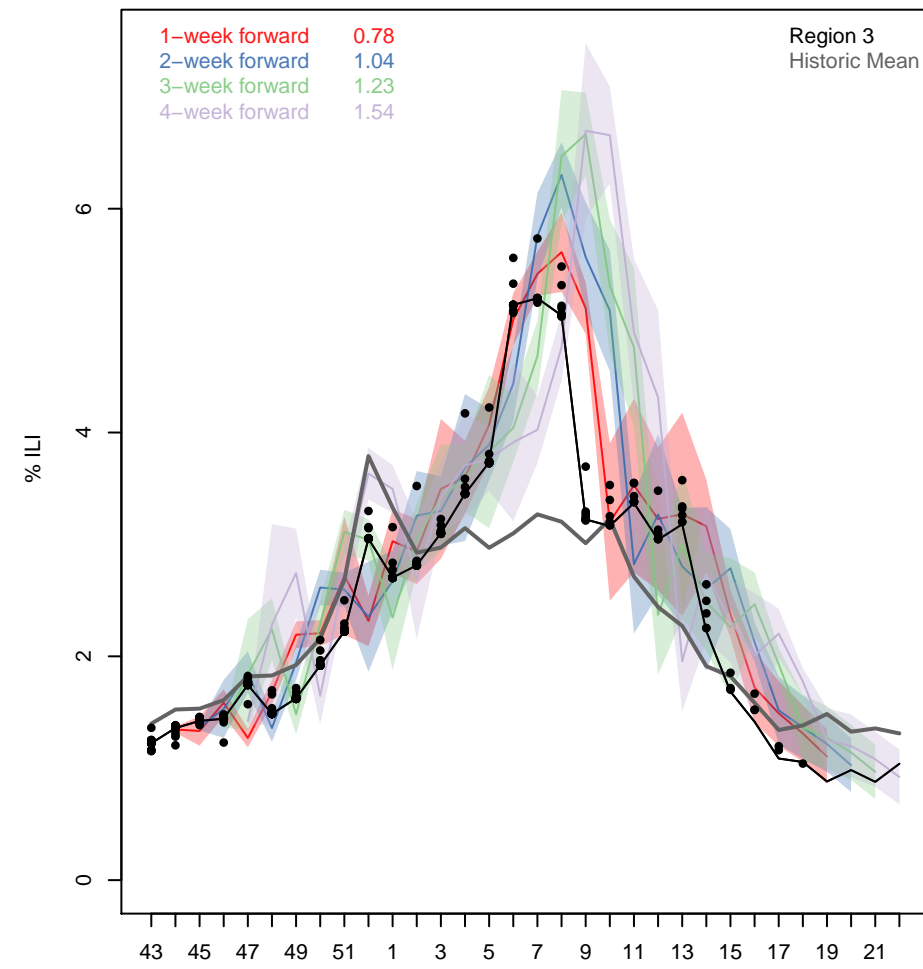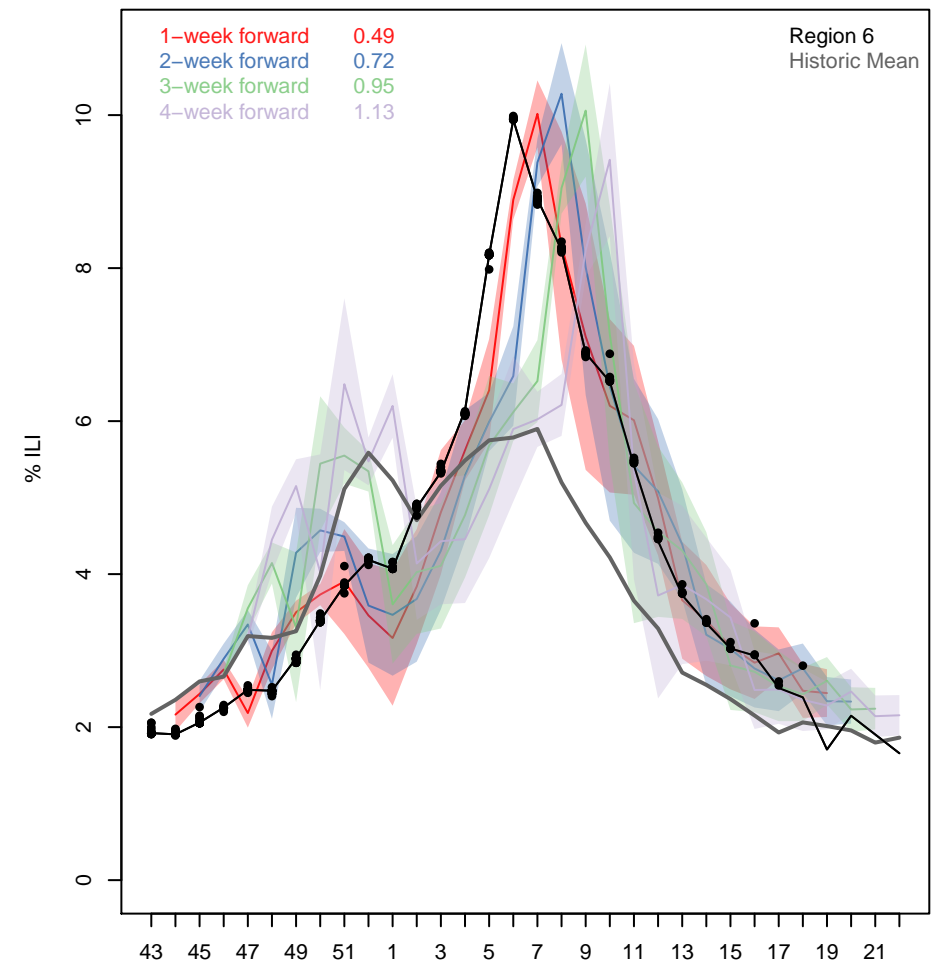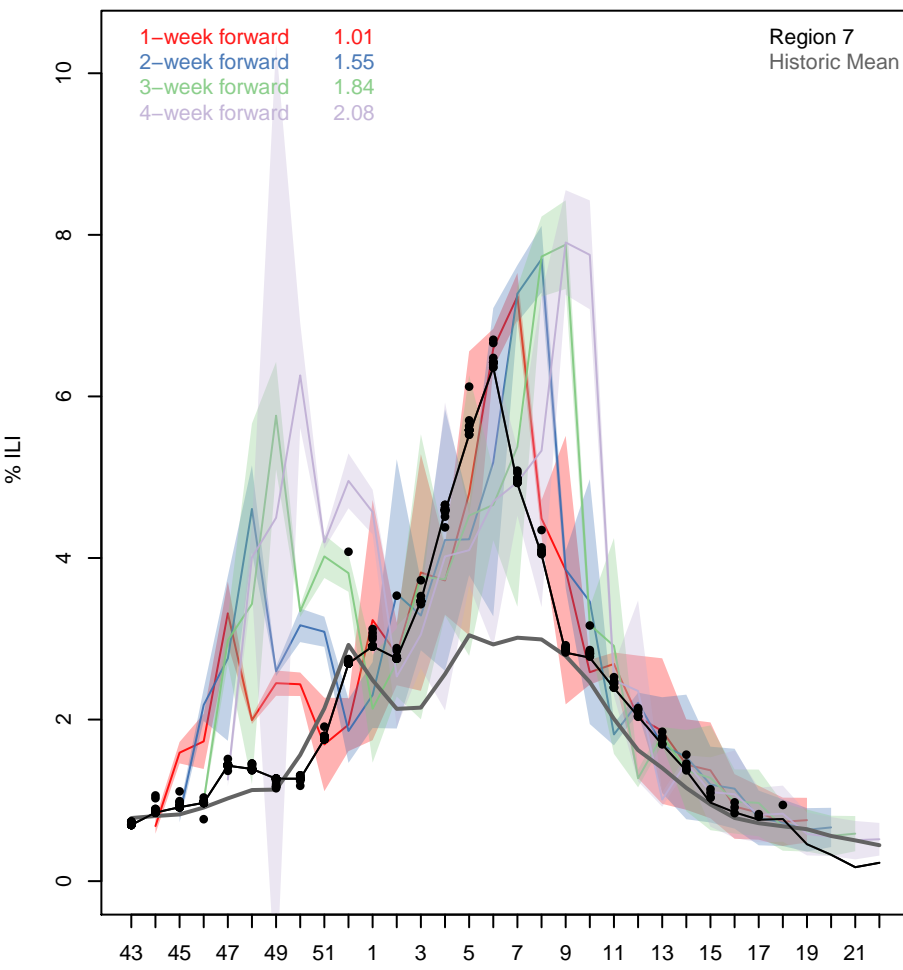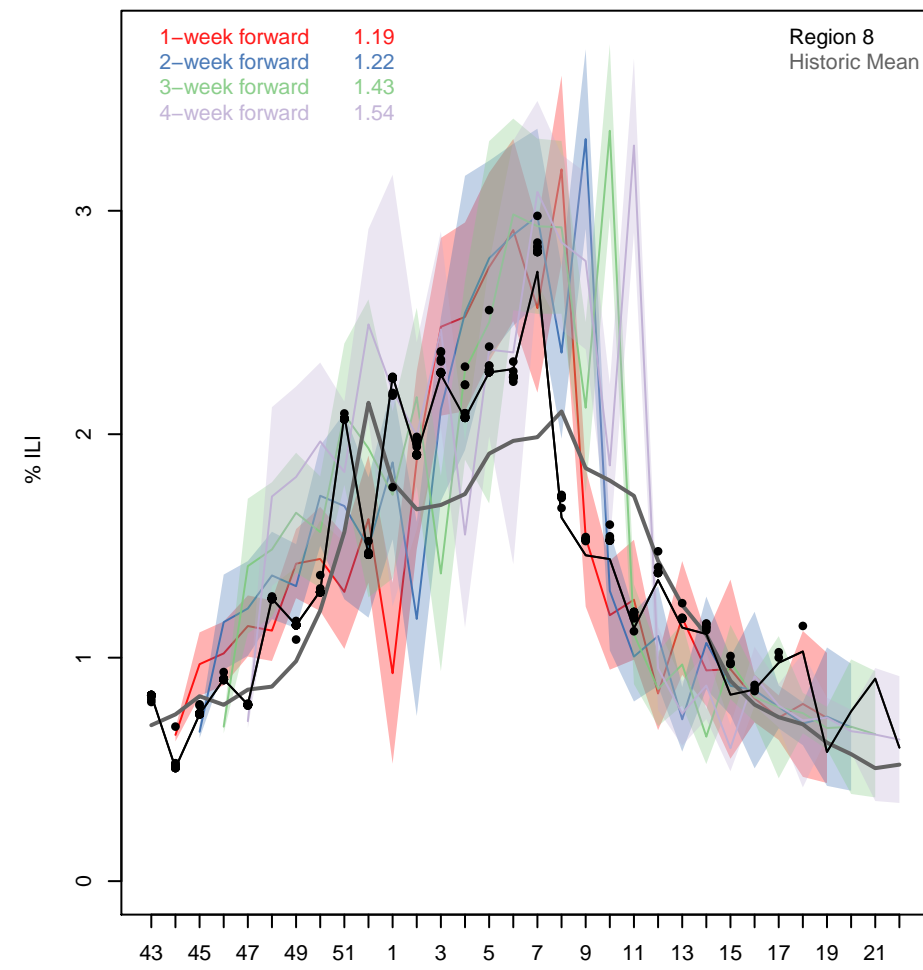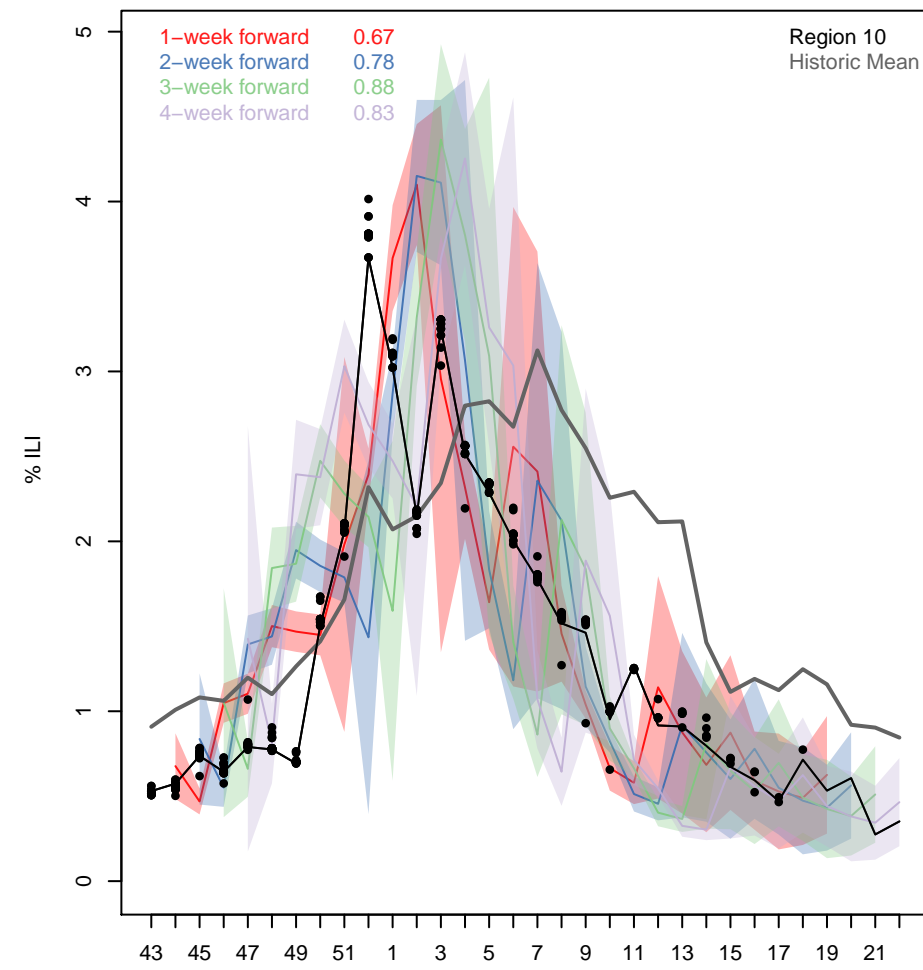

Supplement: S2 Fig — Final season CDC reported (black line), reported during the season (black circles) and predicted %ILI (colored bands) as a function of epidemic week for seven of the ten HHS regions not shown in the main text panel. In each panel the four colored shaded bands denote our n-week forward prediction (n = 1, 2, 3, 4), and the gray line denotes the historic average. The average relative error (measured with respect to the error of the historic NULL model) is indicated for each of the four prediction horizons in the legend. See main text and Fig 2 for more details. (PDF) [file pcbi.1007013.s002.pdf]

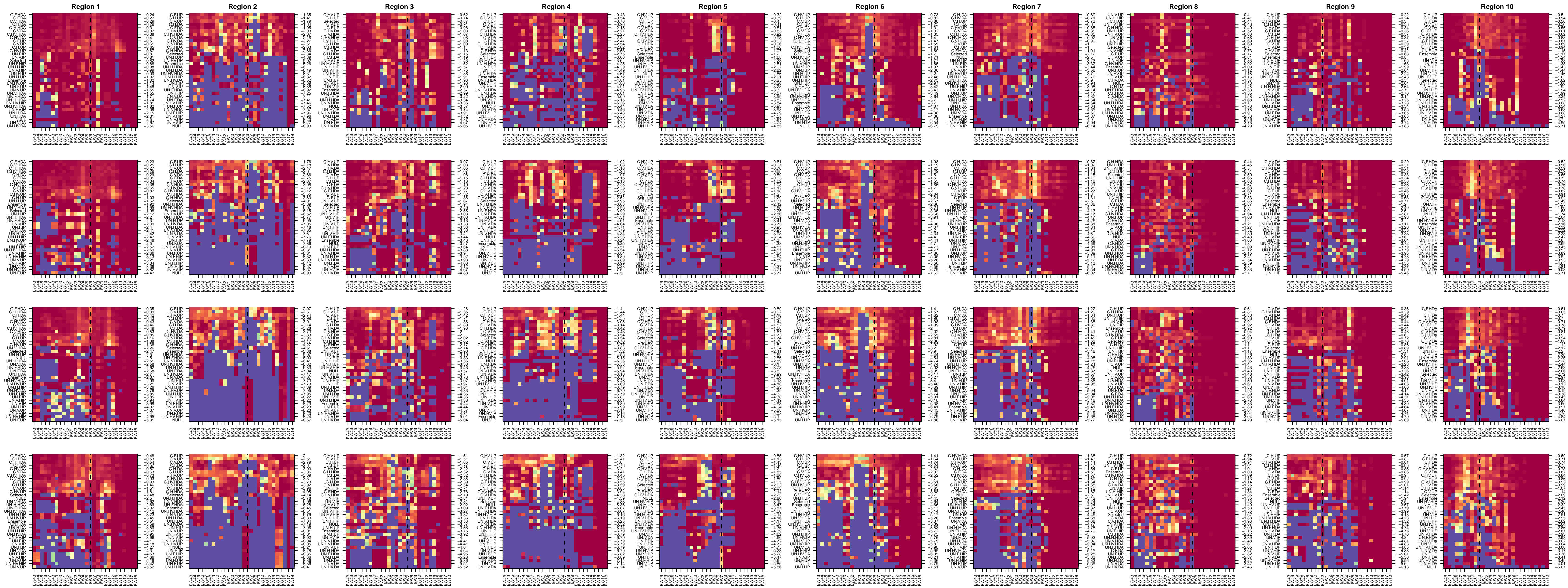

Supplement: S3 Fig — As in the right columns of Fig 4 but for the ten HHS regions. Each columns denotes an HHS region and going down a columns we move from 1 to 2, 3 and 4 weeks forecasts. In the method/model labels (y-axis): UN- uncoupled and C-coupled. H, V, HV and F denote the four models for the force of infection: Humidity only, vacation only, both and fixed. The prior models are: uniform prior (UP), informed prior (IP), heated informed prior (HIP), data augmentation (DA), and heated data augmentation (HDA). Selected, is what we selected each week, the NULL model is the historic average and the ensemble is the average of the 32 model variants. Models are arranged based on their overall performance during the entire season (numbers on the right y-axis) from best (top) to worst (bottom). For all ten regions, and in agreement with the results for the nation, the coupled procedure performs better than the uncoupled. For nearly all regions and forecasts lead times the selected option does better than the ensemble model. To view this Figure better please use the ‘zoom’ tool when opening it with a PDF viewer. (PDF) [file pcbi.1007013.s003.pdf]

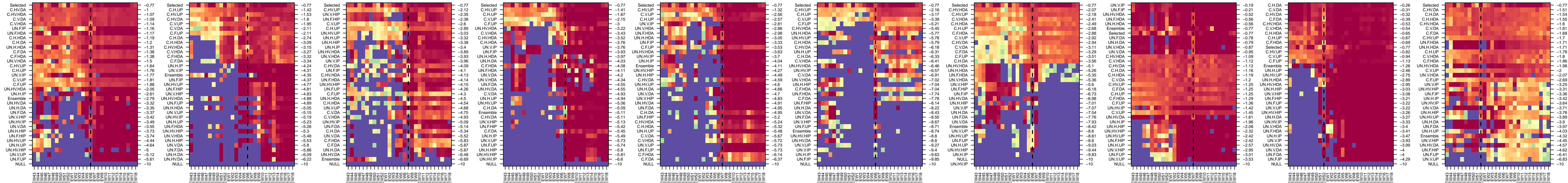

Supplement: S4 Fig — Top, middle and bottom rows are onset week, peak week and peak intensity. The NULL result is calculated using the historic mean regional profile. The ensemble mode is the average of the 32 model variants. For all three targets, and all ten regions, the coupled method does better than the uncoupled with the details of the prior and force of infection models depending on the region. For nearly all targets and regions the selected model does better than the ensemble model. To view this Figure better please use the ‘zoom’ tool when opening it with a PDF viewer. (PDF) [file pcbi.1007013.s004.pdf]

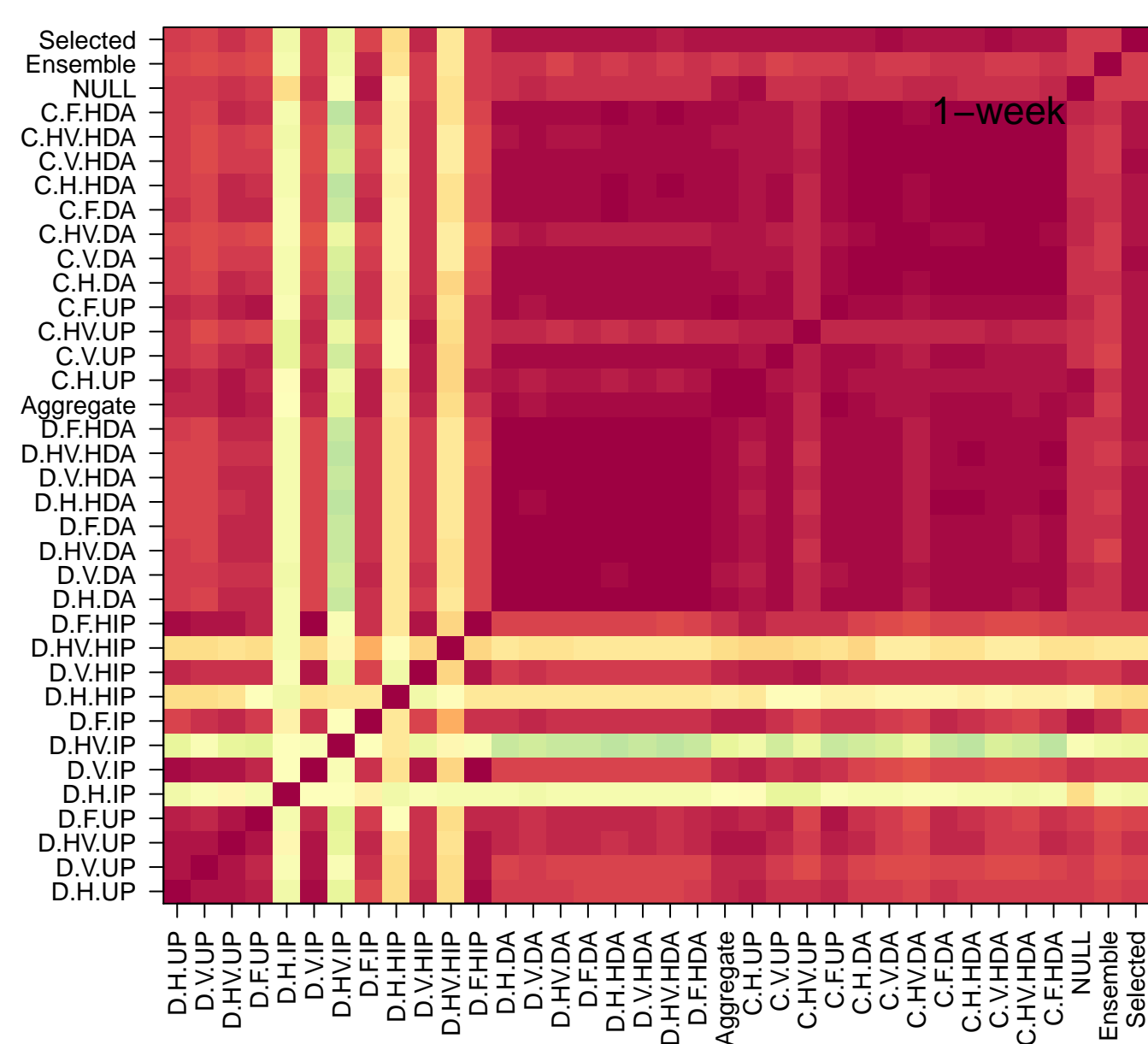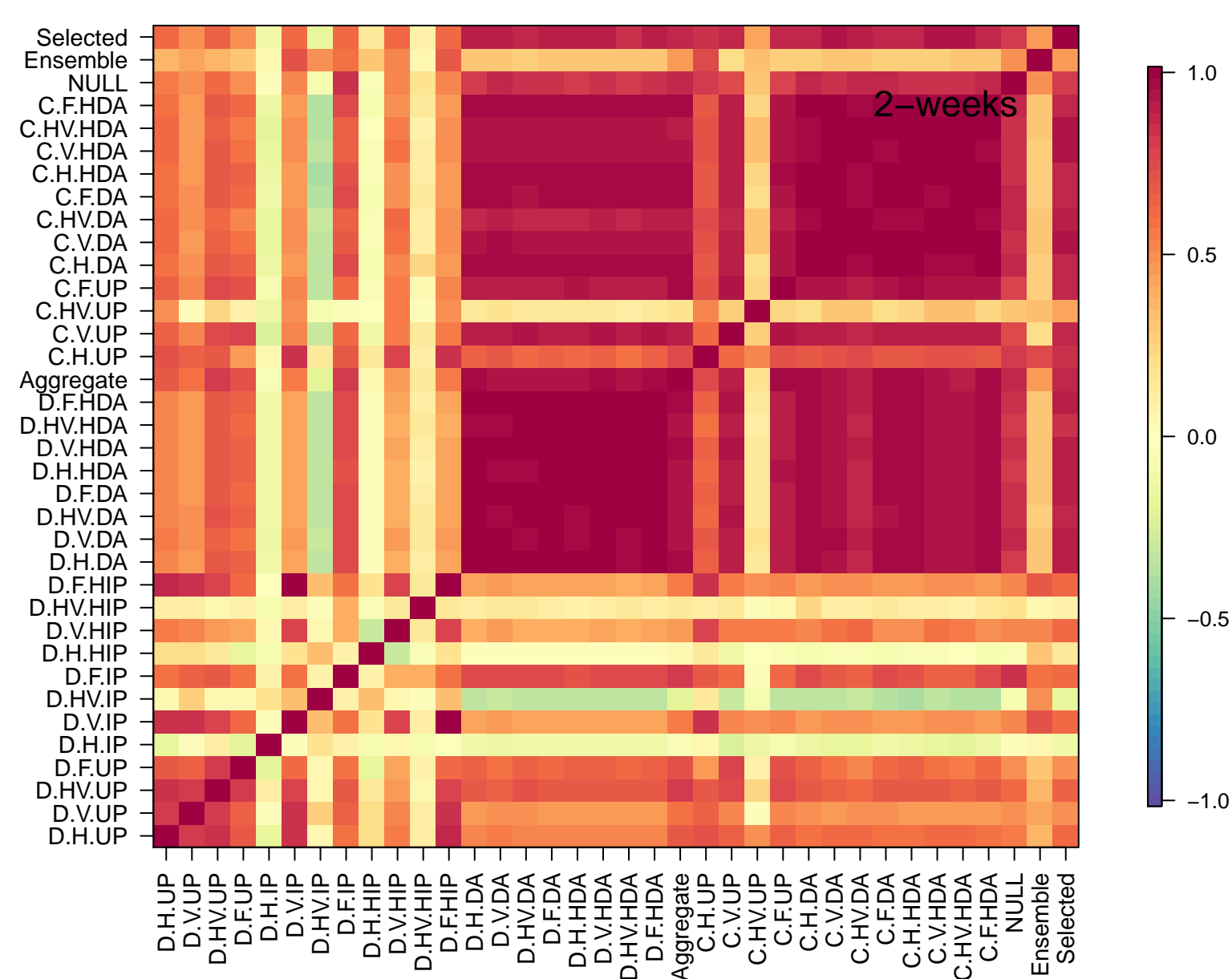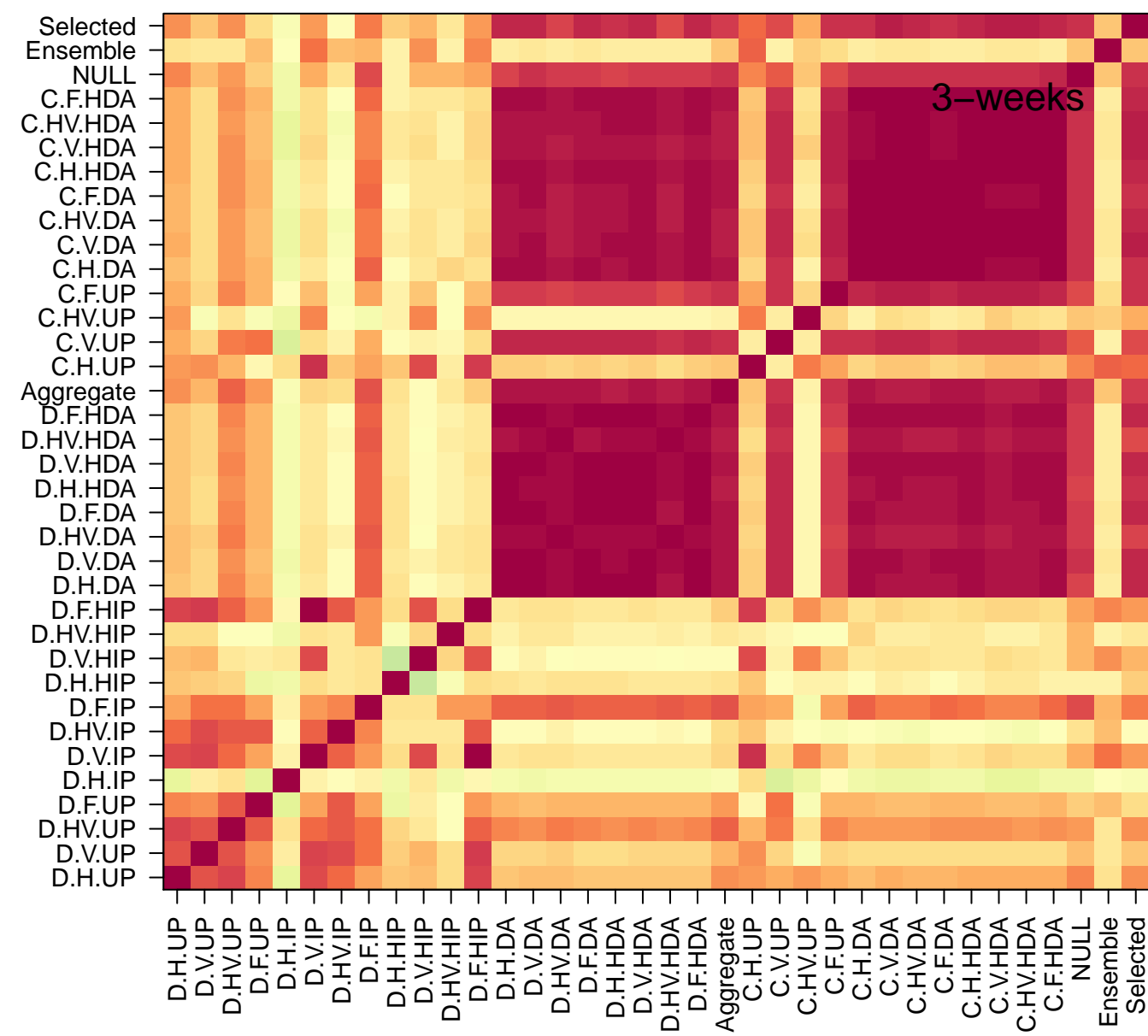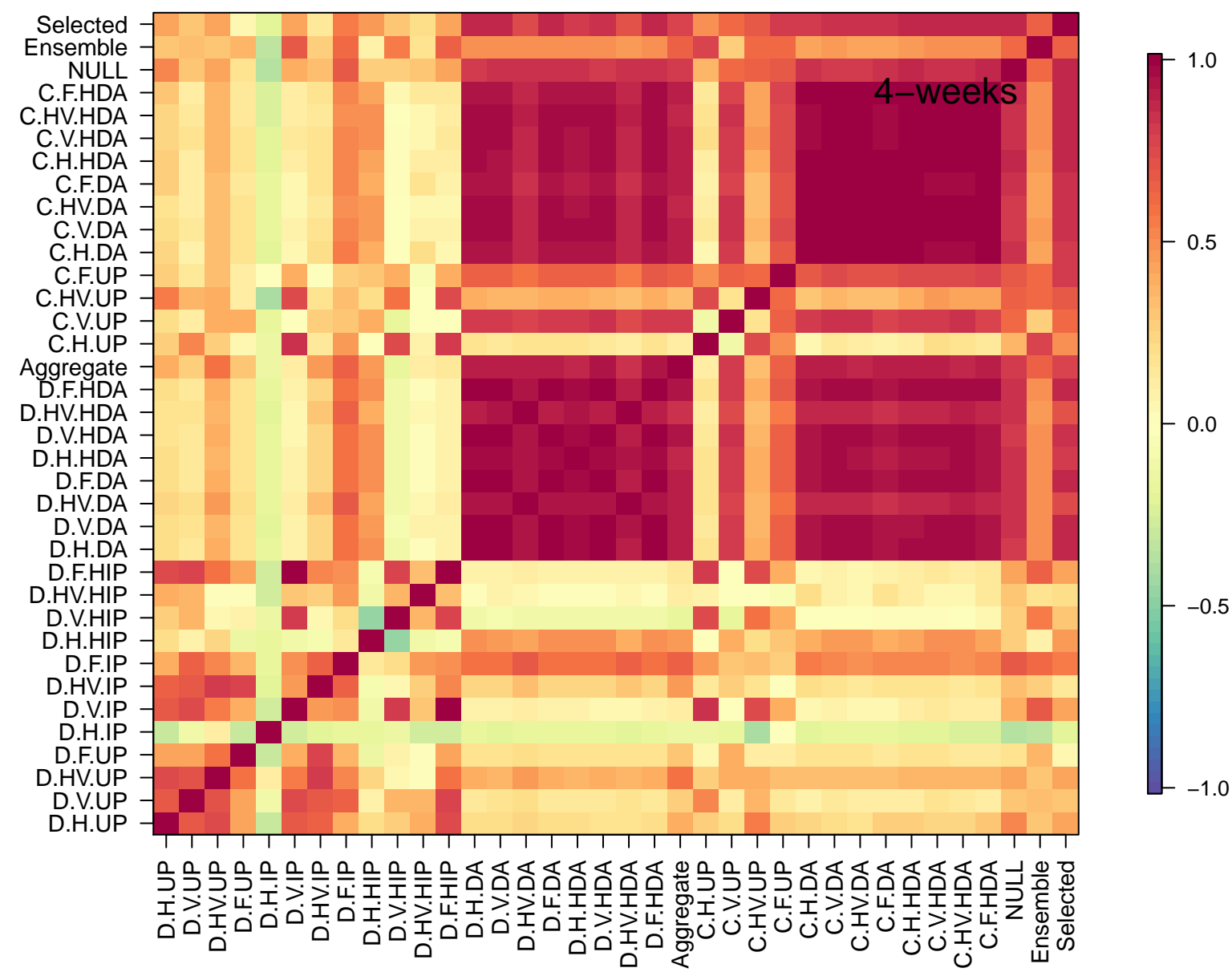

Supplement: S5 Fig — The Pearson correlation between the 1-, 2-, 3- and 4-week forward nation forecast calculated using all models and all 28 weeks of the 2016-17 CDC challenge. For the 1 − 4 weeks forward forecasts the correlation within coupled models and uncoupled models is greater than between uncoupled and coupled. As the prediction horizon increases the correlation between models decreases significantly, but it remains high for the coupled models. (PDF) [file pcbi.1007013.s005.pdf]

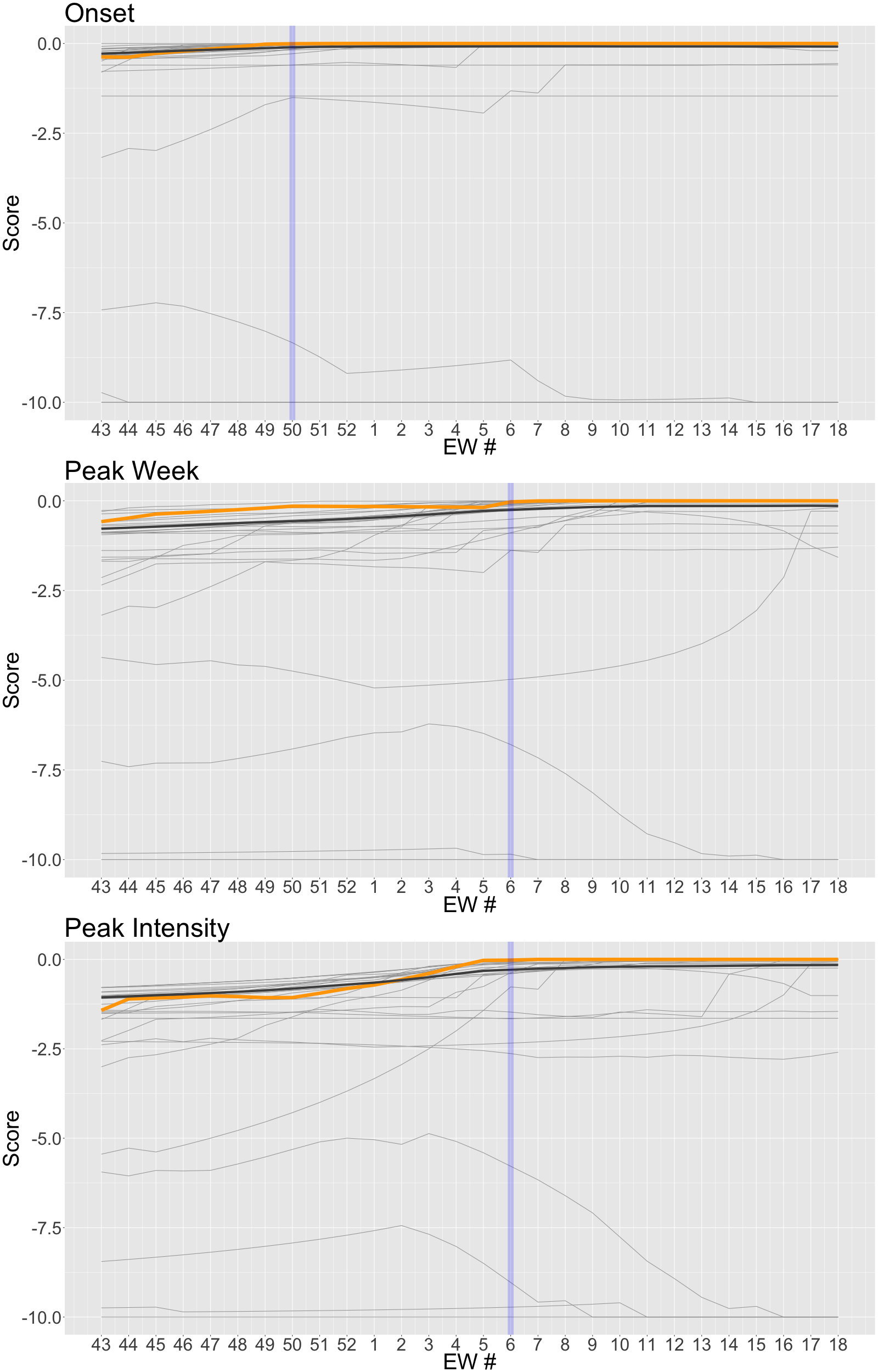

Supplement: S6 Fig — Top, middle and bottom rows are onset week, peak week and peak intensity. Each light gray line denotes one of the 29 submissions to the CDC challenge, the black line in the unweighted average of these submissions and the orange line is that of the DICE model submission. Here, the weekly score is calculated as a reverse rolling average where the first week (EW 43) is the average of all 28 weeks of the challenge, the second week averages the score from week 2 (EW 44) onward etc. This reverse rolling average highlights the gradual improvement of the DICE submission. The vertical blue line marks the season onset week (top panel) and peak week (middle and bottom) panels. (PNG) [file pcbi.1007013.s006.png]

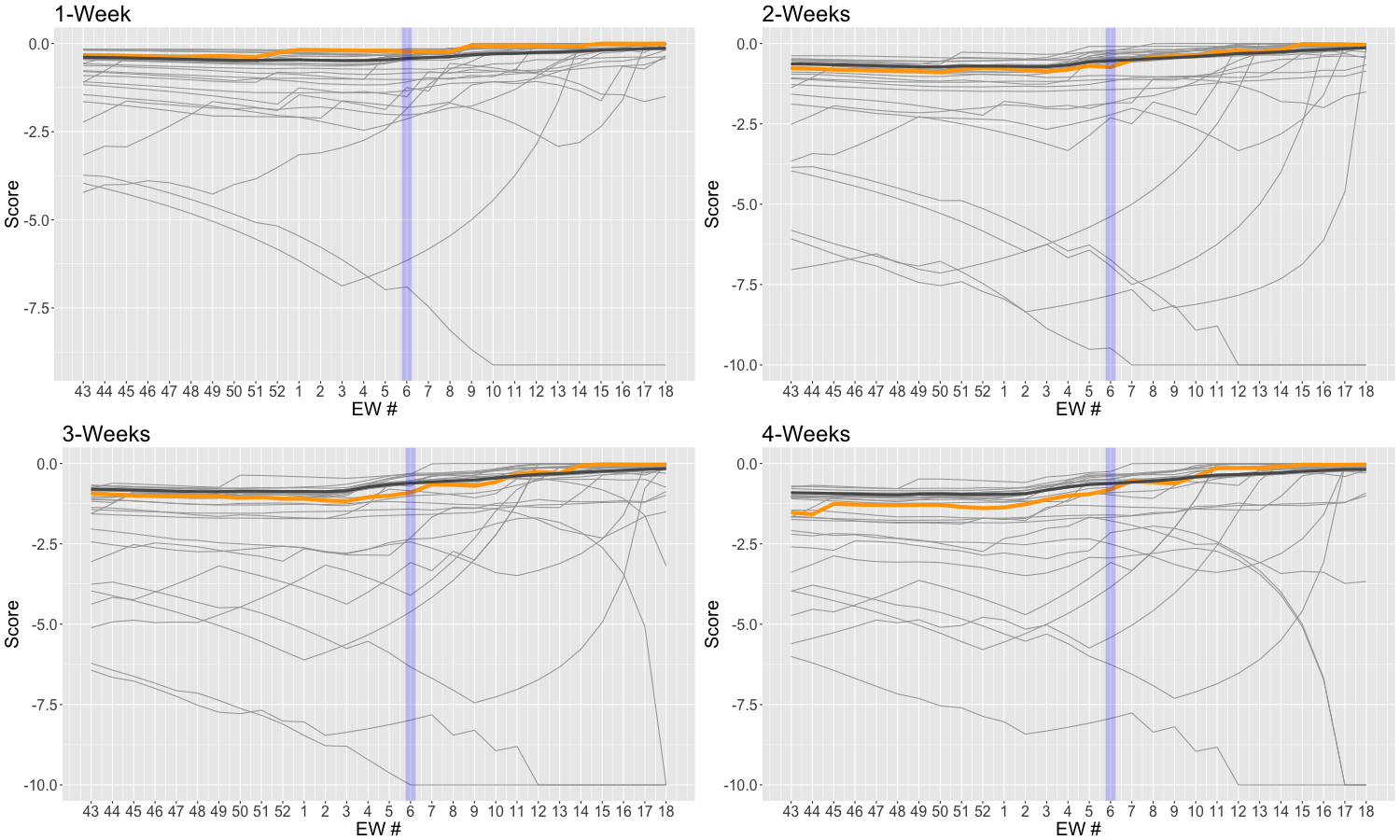

Supplement: S7 Fig — Similar to S6 Fig, but for the 1- to 4- week forward forecast. (PNG) [file pcbi.1007013.s007.png]
