# Supplementary material for: Forecasting national and regional influenza-like illness for the USA
Source: PLoS Comput Biol. 2019 May 23;15(5):e1007013. doi: 10.1371/journal.pcbi.1007013 (PMC6557527; doi:10.1371/journal.pcbi.1007013)
Supplement: S1 Text — (PDF) [file pcbi.1007013.s008.pdf]

S1 Text–Details of the parametric dependence of the force of infection on specific humidity

Eq. 11 allows the transmission rate to depend on time using three terms. The first time dependent term, $F_1(t)$, allows for a dependence of the transmission rate on specific-humidity. In temperate regions specific humidity has a seasonal oscillation with a minimum in the winter and a maximum in the summer. We follow Shaman et al. [1] and relate the local SH, $q_j(t)$, to the reproduction number as:

$$\beta_j(t) = \frac{R_j^0}{T_g} \cdot F_1(t) = R_0 \times [1 + \Delta_R \cdot \mathrm{e}^{-a \cdot q_j(t)}]$$

In the above equation, and unlike the work published by others, the values of the parameters a and Δ_R are fitted. The effect of specific humidity can be combined with that of school vacation which is discussed in the following sub-section.

References

[1] Shaman J, Pitzer VE, Viboud C, Grenfell BT, Lipsitch M. Absolute Humidity and the Seasonal Onset of Influenza in the Continental United States. PLoS Biol. 2010;8(2):1–13.
