# Supplementary material for: Forecasting national and regional influenza-like illness for the USA
Source: PLoS Comput Biol. 2019 May 23;15(5):e1007013. doi: 10.1371/journal.pcbi.1007013 (PMC6557527; doi:10.1371/journal.pcbi.1007013)
Supplement: S2 Text — (PDF) [file pcbi.1007013.s009.pdf]

S2 Text–Details of the parametric dependence of the force of infection on school vacation schedule

The second term in Eq. 11 allows the transmission rate to depend on the weekly school vacation schedule ($p_j(t)$) and we implement it as:

$$\beta_j(t) = \frac{R_j^0}{T_g} \cdot F_2(t) = R_0 \times [1 - \alpha \cdot p_j(t)]$$

DICE fits the effect of school closure by optimizing the parameter α, which is in the range $0-1$. Larger values of α indicate a more significant lowering of $\beta_j(t)$ as a result of planned school vacations. Conversely, small values of α indicate that the school vacation schedule is not an important factor in determining the ILI profile. The effect of school vacation can be combined with that of specific humidity, i.e. $\beta_j(t) = \frac{R_j^0}{T_g} \cdot F_1(t) \cdot F_2(t)$.
